# A novel method for noninvasive quantification of fractional flow reserve based on the custom function

**DOI:** 10.3389/fbioe.2023.1207300

**Published:** 2023-08-30

**Authors:** Honghui Zhang, Xiaorui Song, Rile Wu, Na Li, Qianwen Hou, Jinjie Xie, Yang Hou, Aike Qiao

**Affiliations:** ^1^ Key Laboratory of Intelligent Manufacturing Technology, College of Engineering, Inner Mongolia Minzu University, Tongliao, China; ^2^ Faculty of Environment and Life, Beijing University of Technology, Beijing, China; ^3^ School of Radiology, Shandong First Medical University and Shandong Academy of Medical Sciences, Tai’an, China; ^4^ Department of Neurology, Tong Liao City Hospital, Tongliao, China; ^5^ Beijing Anzhen Hospital, Capital Medical University, Beijing, China; ^6^ Department of Echocardiography, Jiahui International Hospital, Shanghai, China; ^7^ Shengjing Hospital, China Medical University, Shenyang, China

**Keywords:** boundary condition setting, noninvasive quantification, fractional flow reserve, steady-state numerical simulations, custom function

## Abstract

Boundary condition settings are key risk factors for the accuracy of noninvasive quantification of fractional flow reserve (FFR) based on computed tomography angiography (i.e., FFR_CT_). However, transient numerical simulation-based FFR_CT_ often ignores the three-dimensional (3D) model of coronary artery and clinical statistics of hyperemia state set by boundary conditions, resulting in insufficient computational accuracy and high computational cost. Therefore, it is necessary to develop the custom function that combines the 3D model of the coronary artery and clinical statistics of hyperemia state for boundary condition setting, to accurately and quickly quantify FFR_CT_ under steady-state numerical simulations. The 3D model of the coronary artery was reconstructed by patient computed tomography angiography (CTA), and coronary resting flow was determined from the volume and diameter of the 3D model. Then, we developed the custom function that took into account the interaction of stenotic resistance, microcirculation resistance, inlet aortic pressure, and clinical statistics of resting to hyperemia state due to the effect of adenosine on boundary condition settings, to accurately and rapidly identify coronary blood flow for quantification of FFR_CT_ calculation (FFR_U_). We tested the diagnostic accuracy of FFR_U_ calculation by comparing it with the existing methods (CTA, coronary angiography (QCA), and diameter-flow method for calculating FFR (FFR_D_)) based on invasive FFR of 86 vessels in 73 patients. The average computational time for FFR_U_ calculation was greatly reduced from 1–4 h for transient numerical simulations to 5 min per simulation, which was 2-fold less than the FFR_D_ method. According to the results of the Bland-Altman analysis, the consistency between FFR_U_ and invasive FFR of 86 vessels was better than that of FFR_D_. The area under the receiver operating characteristic curve (AUC) for CTA, QCA, FFR_D_ and FFR_U_ at the lesion level were 0.62 (95% CI: 0.51–0.74), 0.67 (95% CI: 0.56–0.79), 0.85 (95% CI: 0.76–0.94), and 0.93 (95% CI: 0.87–0.98), respectively. At the patient level, the AUC was 0.61 (95% CI: 0.48–0.74) for CTA, 0.65 (95% CI: 0.53–0.77) for QCA, 0.83 (95% CI: 0.74–0.92) for FFR_D_, and 0.92 (95% CI: 0.89–0.96) for FFR_U_. The proposed novel method might accurately and rapidly identify coronary blood flow, significantly improve the accuracy of FFR_CT_ calculation, and support its wide application as a diagnostic indicator in clinical practice.

## 1 Introduction

Coronary heart disease, including coronary stenosis, has become the disease with the highest mortality rate worldwide (Bruyne et al., 2014; Zhang et al., 2021a; Li X. J. et al., 2021). Currently, pressure field-based fractional flow reserve (FFR) is the gold standard for clinical diagnosis of myocardial ischemia severity caused by coronary stenosis (Cesaro et al., 2018; Zhang et al., 2021b). For patients with invasive FFR, complex invasive surgical operations are often required, with potential risks and high measurement costs during catheter insertion. Many studies have been devoted to exploring the noninvasive alternatives to invasive FFR.

Computed tomography angiography (CTA)-derived fractional flow reserve (FFR_CT_) is a viable alternative method for the noninvasive calculation of FFR. FFR_CT_ combines a coronary model (typically obtained from computed tomography angiography images) and computational fluid dynamics (CFD), to visualize the pressure field across the coronary tree (Taylor et al., 2013), and to further assess the severity of myocardial ischemia. However, previous clinical studies have shown that the diagnostic accuracy of FFR_CT_ calculation obtained based on a one-dimensional model (1D model) commonly used in clinics is still insufficient compared with the method proposed by Taylor (84.3%). Coenen et al. detected invasive FFR and FFR_CT_ calculated by the 1D model in 144 vessels with intermediate coronary stenosis, and the accuracy of FFR_CT_ calculation was 71.5% (Coenen et al., 2015). Subsequently, the accuracy of FFR_CT_ calculation was slightly improved (75%) in a study by Coenen and colleagues, who performed invasive FFR and FFR_CT_ calculated by 1D model on 203 vessels with coronary stenosis (Coenen et al., 2016). In another study, invasive FFR and FFR_CT_ calculations were performed on 23 vessels with coronary stenosis, and the accuracy of FFR_CT_ calculation was 78% (Geer et al., 2016). In addition, Baumann et al. performed invasive FFR and FFR_CT_ calculated by a 1D model for 36 vessels with coronary stenosis, and the Pearson correlation coefficient was only 0.74 (Baumann et al., 2015). Of these, the insufficiency of the 1D model in the accuracy of FFR_CT_ calculation is that it only captures the variation of the vascular pressure along the axial direction, as well as the impact of the minimum stenotic diameter and stenotic length on the vascular pressure distribution, while ignoring the impact of other characteristics of stenotic structures (e.g., eccentric, continuous stenosis) on vascular pressure distribution. Based on this situation, it is necessary to develop a novel method to improve the accuracy of FFR_CT_ calculation.

Many clinical studies have reported that, in addition to the minimum stenotic diameter and stenotic length, the characteristics of the stenotic structure have a significant impact on the accuracy of FFR_CT_ calculation. For example, Modi et al. proved the significant impact of serial coronary stenosis on changes in FFR_CT_ calculation (Modi et al., 2019); Rajkumar et al. demonstrated that diffuse stenosis has a significant impact on FFRCT calculation (Rajkumar et al., 2021). Zaman et al. showed that changes in lesions located in bifurcated vessels had a significant impact on changes in FFR_CT_ calculation (Zaman et al., 2021). In addition, many clinical studies have also shown that a three-dimensional (3D) model of the coronary artery contains more model characteristics of stenotic structures in CFD simulation, and has higher accuracy of FFR_CT_ calculation (84.3%) (Min et al., 2012; Gaur et al., 2013; Nørgaard et al., 2014). So the 3D spatial structure of the coronary artery should be comprehensively considered to improve the accuracy of FFR_CT_ calculation. However, since only the change of the outlet microcirculation resistance caused by the effect of adenosine was considered in the above studies, the boundary condition settings of FFR_CT_ calculation limited the calculation accuracy below 84.3%, and FFR_CT_ calculation based on transient numerical simulations required significant time (1–4 h per simulation). Therefore, to further improve the accuracy of FFR_CT_ calculation and reduce the computational time cost, it is necessary to set the boundary condition of FFR_CT_ calculation according to the clinical statistics of hyperemia state due to the effect of adenosine, including decreased inlet aortic pressure, decreased microcirculation resistance, and increased blood flow, and considering the interaction of stenotic resistance, microcirculation resistance and inlet aortic pressure to identify coronary blood flow to quantify FFR_CT_ calculation under steady-state numerical simulations.

In this study, to improve the accuracy of FFR_CT_ calculation and reduce the computational time cost, we developed a novel method (FFR_U_) based on a 3D model of the coronary artery, integrating boundary condition settings with clinical statistics of hyperemia state, and the custom function taking into account the interaction of stenotic resistance, microcirculation resistance and inlet aortic pressure to identify coronary blood flow. Subsequently, we tested the diagnostic accuracy of FFR_U_ by comparison with existing methods (CTA, coronary angiography (QCA), and diameter-flow method for calculating FFR (FFR_D_)) based on the invasive FFR of 86 vessels in 73 patients.

## 2 Methods

### 2.1 Geometry model of coronary artery

The study was conducted at Beijing Anzhen Hospital of the Capital Medical University and Shengjing Hospital of China Medical University. The inclusion criteria for patients in this study had complete clinical data and underwent CTA, quantitative coronary arteriography (QCA), transthoracic echocardiography, and invasive FFR measurement within 30 days. The exclusion criteria for this study were as follows: 1) Poor quality of CT images; 2) Unstable angina; 3) Prior myocardial infarction; 4) Prior percutaneous coronary intervention or coronary artery bypass grafting; 5) Diffuse coronary stenosis; 6) Left ventricular ejection fraction (LVEF) < 50%; 7) Severe valve disease; 8) Atrial fibrillation; 9) Severe microcirculation disturbance; 10) Allergy to contrast agents and vasodilators. Finally, 73 patients with stable angina were enrolled between August 2013 and April 2019 in this study. The quality of CT images in all patients was examined and assessed by two experienced radiologists. Cardiac output and left ventricular ejection fraction in all patients were measured and calculated by two experienced echocardiographers based on the structural characteristics of the heart (Li G. Y. et al., 2021).

The measurement of invasive FFR relied on three steps: 1) Adenosine (140 μg/kg/min) was administrated through intravenous infusion to induce maximum hyperemia of the coronary artery; 2) We obtained pressure waveforms of aortic pressure and distal arterial pressure using pressure wire measurement; 3) We further calculated the invasive FFR for the ratio of the mean pressure at a cross-Section 3 cm downstream of the stenosis (P_d_) to the aortic pressure (P_a_) at least three cardiac cycles (Taylor et al., 2013; Zhang et al., 2021a). Patient informed consent was waived due to the retrospective nature of the study.

**FIGURE 1:**
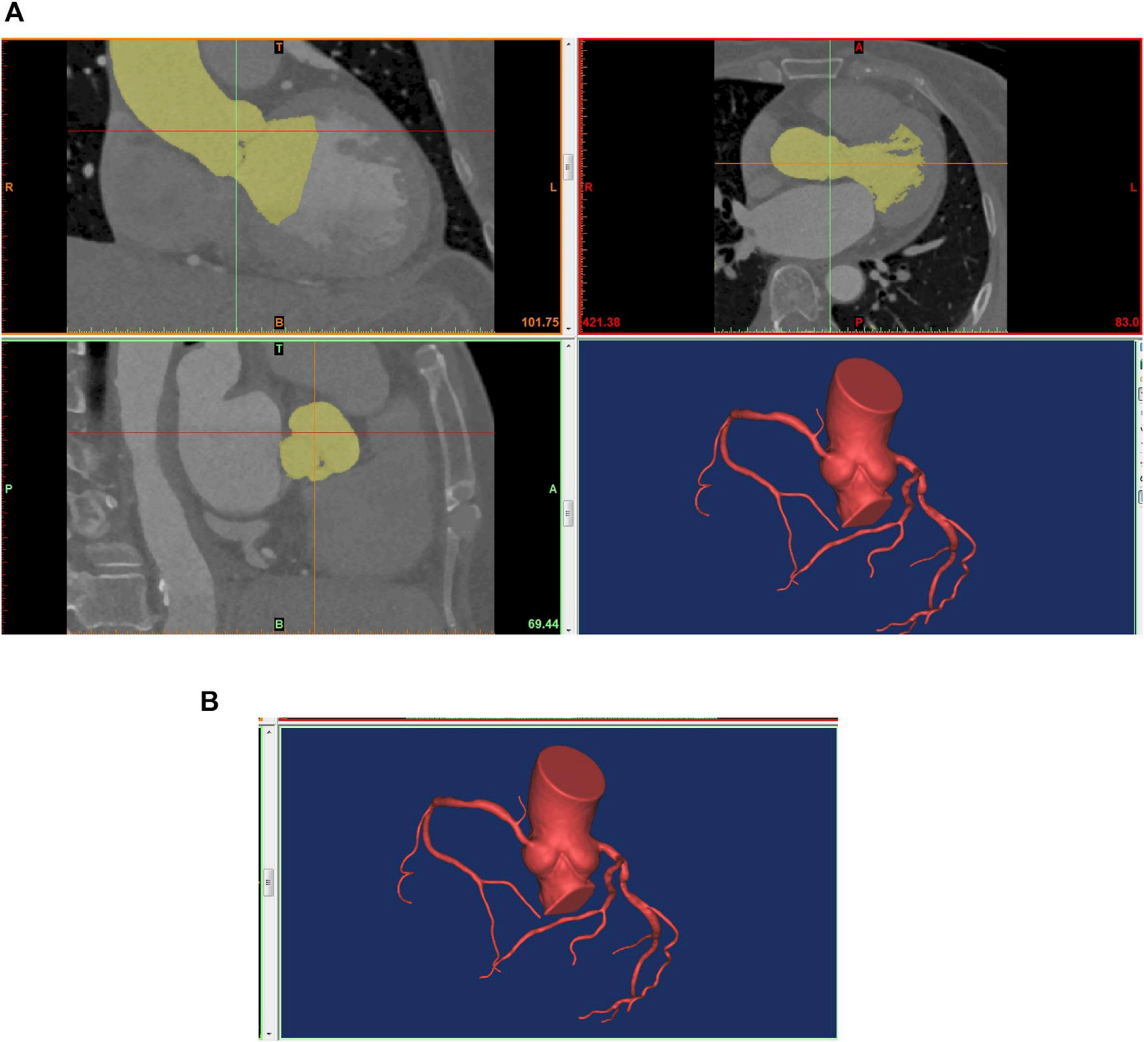
Technical flow chart of 3D reconstruction of the coronary artery. **(A)** 3D reconstruction based on CTA; **(B)** The reconstructed model for ANSYS CFX.

Based on the patient’s CT image information, the 3D model of the coronary artery was reconstructed using the commercial software MIMICS (Materialise, Leuven, Belgium). The coronary arteries with diameters larger than 1 mm were reconstructed. Subsequently, the coronary reconstructed noise was removed using the Freeform tool (Artec 3d, Luxembourg). And the reconstructed coronary surfaces were further repaired and smoothed using the commercial software GEOMAGIC (Geomagic, Research Triangle Park, North Carolina). Then, the coronary centerline was identified to calculate the diameter and length of the vessel using the MIMICS software. GEOMAGIC was used to divide the reconstructed coronary surfaces into curved surfaces for hemodynamics simulation. Later, the inlet and outlet of the reconstructed coronary artery were cut into planes, and boundary conditions for pressure or mass flow were loaded using the SOLIDWORKS software (Dassault Systemes, Waltham, Massachusetts). Finally, the reconstructed coronary artery was imported into ANSYS CFX (ANSYS Corporation, Canonsburg, Pennsylvania) for CFD simulation. The process of 3D reconstruction of the coronary artery was shown in Figure 1.

### 2.2 Distribution of coronary branch blood flow at resting state

Based on the available literature and clinical reports, coronary blood flow was 4% of cardiac output (Kim et al., 2010), and we can calculate coronary blood flow by transthoracic echocardiography measurement. Based on Poiseuille’s and the law of minimum energy dissipation, the allometric scaling law between coronary morphological and functional parameters was quantified using *in vitro* experiments (Taylor et al., 2013; Zhang et al., 2021b). We further established the distribution law of blood flow of the coronary branch on the basis of the form-follow-function scaling law described by Huo, in which the blood flow of the parent and daughter branches of the coronary artery was related to their respective effective diameters (Huo et al., 2012). These findings had a good consistency with the earlier *in vitro* experimental results of Zhou (Zhou et al., 1999). The allometric scaling law between blood flow and the diameter of the coronary branch followed a power-law relationship as shown in Eq. 1.

$$\frac{Q_{s}}{Q_{\max}}={\left(\frac{D_{s}}{D_{\max}}\right)}^{\frac{7}{3}}$$ (1)

In Eq 1, *Q*_*max*_ and *D*_*max*_ represent the blood flow and diameter of the parent branch, and *Q*_*s*_ and *D*_*s*_ represent the blood flow and diameter of the daughter branch.

### 2.3 Calculation of microcirculation resistance at resting state

Broadly speaking, vessel resistance was calculated using morphological parameters (cross-sectional area and length) according to Poiseuille’s law. The equation followed a power-law relationship, as shown in Eq 2.

$$R=\frac{8\pi \mu L}{A^{2}}$$ (2)

$$A=\frac{A_{inlet}+A_{outlet}}{2}$$ (3)

In Eq 2, *R*, *L*, and *A* represent the resistance, length, and cross-section area of the coronary branch, and *μ* represents the dynamic viscosity of blood flow. In Eq 3, *A*_*inlet*_ and *A*_*outlet*_ represent the cross-section area at the inlet and outlet of the coronary branch.

Ohm’s law describes the relationship between current, resistance, and voltage in a circuit. The blood flow, resistance, and pressure drop along the coronary arteries represent the corresponding parameters, respectively (Li B. et al., 2021). So the pressure drop ( ∆P ) along the coronary arteries was described by Eq 4.

$$∆P={RQ}_{s}$$ (4)

Systolic and diastolic blood pressure measurements were performed during the statistical process of clinical data. The calculation of mean blood pressure was described by Eq 5.

$$P_{MBP}=\frac{\left(P_{sp}+2P_{dp}\right)}{3}$$ (5)

In Eq 5, $P_{MBP}$ , $P_{sp}$ , and $P_{dp}$ represent mean blood pressure, systolic blood pressure, and diastolic blood pressure, respectively.

Based on the mean blood pressure and pressure drop along the coronary arteries, the inlet pressure of the microcirculation was calculated stepwise from the proximal to the distal end of coronary arteries using Eqs 4, 5. The microcirculation resistance was calculated using Eq 6.

$$R_{i}=\frac{P_{i}}{Q_{s}}$$ (6)

In Eq 6, $R_{i}$ and $P_{i}$ represent the resistance and inlet pressure of the microcirculation, and *i* represents the number of coronary branches, *i = 1, 2, 3…16*.

### 2.4 Identifying boundary conditions for coronary inlet and outlet at hyperemia state

Extensive literature and clinical cases have shown that mean blood pressure and microcirculation resistance decrease as the coronary circulation changes from a resting to a hyperemia state (Wilson et al., 1990; Tang et al., 2020). Many pieces of literature have reported that the severity of coronary epicardial stenosis has no affected on minimal microvascular resistance (Asrnoudse et al., 2004; Fearon et al., 2004; Zhang et al., 2016). In this study, mean blood pressure was reduced by 12% (Wilson et al., 1990), and microcirculation resistance was taken to be 0.23 times the resting state to mimic the hyperemia state caused by the effect of adenosine (Wilson et al., 1990).

$$P_{0}=P_{MBP}-0.12P_{MBP}$$ (7)

$$R_{j}=0.23R_{i}$$ (8)

In Eqs 7, 8, $P_{0}$ and $R_{j}$ represent mean blood pressure and microcirculation resistance at hyperemia state, respectively, *i = j = 1, 2, 3…16*.

### 2.5 Identifying the blood flow at the outlet of the coronary branch

Eqs 9, 10 were coded in Fortran by using the user-defined function (UDF) of ANSYS CFX, running on an HP Z8 workstation. Then, the fluid dynamics analysis of the coronary artery was updated with the under-relaxation scheme as formulated in Eqs 9, 10. Eq. 9 until the sum of the pressure gradient of the epicardial coronary and microcirculation resistance matched the inlet pressure at hyperemia, and Eq. 10 until the target residual of the pressure gradient at the outlet of the coronary branch was determined to be 1e-4. Finally, we identified the blood flow at the outlet of the coronary branch under the hyperemia state.

$$Q_{j,new}={\left(1-\alpha \right)Q}_{j,old}+\alpha \left(\frac{P_{j,old}}{R_{j}}\right)$$ (9)

$$P_{j,new}=P_{j,old}+\alpha \left(P_{0}-P_{j,old}+Q_{j,new}R_{j}\right)$$ (10)

In Eqs 9, 10, $P_{j,old}$ , $P_{j,new}$ , $Q_{j,old}$ , and $Q_{j,new}$ represent the pressure and blood flow before and after iterative calculation at the outlet of each coronary branch, respectively, and α represents the under-relaxation factor.

### 2.6 Application of the calculation of FFR_U_ of the coronary stenosis

Blood flow was modeled as a Newtonian fluid. The blood flow state was steady, and the properties of the arterial walls were set to non-slip rigid (Zhang et al., 2014). The density and viscosity of blood flow were set at 1,050 kg/m^3^ and 0.0035 Pa s, respectively (Zhang et al., 2021a). The mesh of the coronary geometry model discretized the computational domain into tetrahedral elements. In this study, the CPU of an HP Z8 workstation is a Dual Intel Xeon Silver 4,210 processor, and the memory of the workstation is 128 GB. The mesh of the geometries was generated by using nonstructural tetrahedron elements. The maximum grid size was 0.23 mm based on the grid independence test. Then, we used the 3D N-S function to calculate the pressure and blood flow field of the coronary arteries.

**FIGURE 2:**
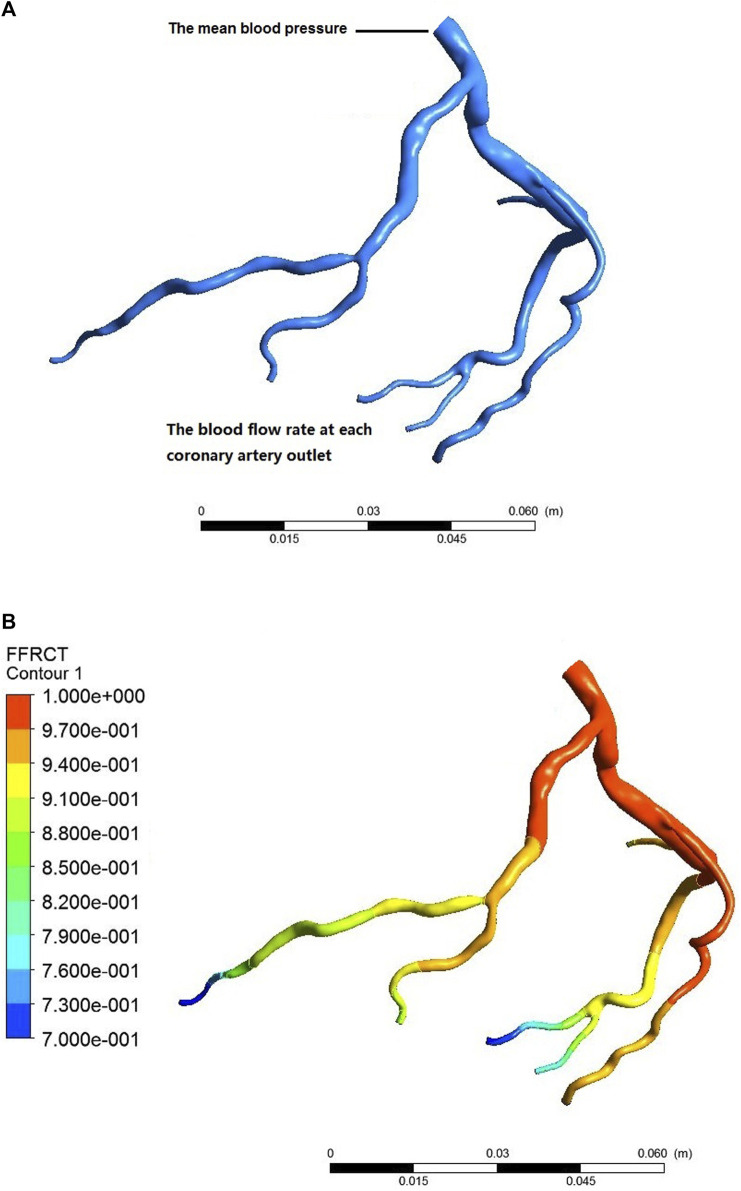
Process of calculating coronary FFR_CT_. **(A)** individualized settings of the boundary conditions; **(B)** CFD simulation postprocessing.

We determined the pressure at the coronary inlet based on the effect of adenosine on mean blood pressure. Then, we can quickly identify the blood flow of each coronary branch by compiling a user-defined function at the outlet of the coronary branch. Later, combining the mean blood pressure as the inlet boundary condition and the blood flow at the outlet of each coronary branch to compile a user-defined function as the outlet boundary condition, we implemented a CFD simulation of the coronary arteries using ANSYS CFX. Finally, we extracted the pressure field of the coronary arteries to calculate FFR_U_, the ratio of the pressure at a cross-Section 3 cm downstream of the stenosis to the aortic pressure, as shown in Figure 2.

### 2.7 Application of the calculation of FFR_D_ of the coronary stenosis

Based on the mean values of aortic diastolic pressure, myocardial mass, and heart rate of the patient, we calculated a patient-specific coronary blood flow rate according to the empirical formula of total coronary blood flow rate. Based on the volume or diameter from CCTA, we achieved patient-specific distribution of the blood flow of the left and right coronary artery. Based on the blood flow of the left and right coronary artery and the distribution rule, we achieved a patient-specific coronary blood flow rate at each terminal branch. Combining the mean value of aortic diastolic pressure as the inlet boundary condition and the coronary blood flow rate at each terminal branch as the outlet boundary condition, we obtained the patient-specific boundary conditions of the fluid dynamics analysis. Based on the fluid dynamics analysis of the coronary artery, we extracted the pressure field of the coronary artery, and we calculated the FFR_D_ as the ratio of the mean pressure at a cross-Section 3 cm downstream of the stenosis to the mean arterial pressure.

In this study, to identify the blood flow at the outlet of the coronary branch and accelerate the calculation convergence, a user-defined function (UDF) was compiled to identify the blood flow and pressure at the outlet of each coronary branch. Then, a user-defined function was used to integrate the interaction of stenotic resistance, microcirculation resistance, and inlet aortic pressure to identify coronary blood flow to quantify FFR_U_ calculation based on boundary conditions of clinical statistics of hyperemia state. In contrast, as for FFR_D_ calculation, the blood flow at the outlet of each coronary branch was distributed step by step along the proximal to the distal blood flow direction based on the hyperemia state, and it ignored the impact of coronary stenosis on the distribution of coronary blood flow.

### 2.8 Statistical analysis

Clinical data analysis included clinical statistics, CTA, QCA, transthoracic echocardiography, and invasive FFR. Continuous and categorical were shown as mean, frequency, and/or percentage, respectively.

To evaluate the diagnostic accuracy of the novel method for calculating FFR_U_, we used the novel method to calculate FFR_U_ for 86 vessels in 73 patients and then compared these data with those derived from existing methods and invasive FFR.

Bland-Altman plot with 95% confidence intervals (CI) was used to evaluate the consistency of the novel method for FFR_U_ calculation and the existing method (FFR_D_) with invasive FFR. Based on the reference value of invasive FFR≤0.8 for the diagnosis of lesion-specific myocardial ischemia, we adopted the metrics of receiver operating characteristic curves (AUC) with 95% (CI), sensitivity, specificity, positive predictive value (PPV), and negative predictive value (NPV), to assess the diagnostic accuracy of FFR_U_ calculation and existing methods (CTA≥50% stenosis, QCA≥50% stenosis and FFR_D_).

## 3 Results

### 3.1 Patient characteristics

The baseline demographics of the patients were shown in Table 1. Of these, more than half of the patients were men (69.86%), and the mean patient age was 59 ± 16 years.

**TABLE 1:** Baseline demographic and clinical characteristics.

	*Study population*	
*Variable*	*Number*	*Percent* (%)
Gender		
Male	51	69.86
Female	22	30.14
Age (years)	59 ± 16	
Body mass index (kg/m^2^)	28.4 ± 5.6	
HR (beat per minute)	68 ± 24	
Mean blood pressure	95.5 ± 10.8	
Cardiac output (L/min)	4.2 ± 1.6	
Left myocardial mass (g)	142.5 ± 46.6	

### 3.2 Measurement of QCA and invasive FFR

QCA and invasive FFR measurements were successfully performed in 86 vessels. The coronary arteries of most patients (79.45%) were right-dominant patterns by QCA. Among the 86 vessels, more than half of the lesions (68.6%) occurred in the left anterior descending artery (LAD), and 67 vessels (77.91%) had luminal stenosis≥50%. Of these, only 32 vessels (37.21%) showed significant ischemia (FFR≤0.8), as shown in Table 2.

**TABLE 2:** Type of coronary artery distribution and the vessels with measured QCA and invasive FFR.

*Variable*	*Number*	*Percent* (%)
Type of coronary artery distribution		
Left dominant pattern	8	10.96
Right dominant pattern	58	79.45
Balanced dominant pattern	7	9.59
Measured FFR vessels	86	
LAD	59	68.6
LCX	19	22.09
RCA	8	9.31
Luminal stenosis≥50%	67	77.91
Invasive FFR≤0.8	32	37.21

LCX, is left circumflex artery; RCA, is right coronary artery.

### 3.3 Calculation of FFR_U_ and FFR_D_

The calculation of FFR_U_ and FFR_D_ was successfully performed on 86 vessels using a HP Z8 workstation. The average computational time for FFR_U_ was significantly reduced, taking only 5 min per simulation, which was 2-fold less than the FFR_D_ method. Table 3 shows the distribution of FFR_D_ and FFR_U_ calculated by the two approaches in 86 vessels. Regarding the calculation of FFR_D_, the numbers 27 and 5 represent that 27 vessels were calculated as having FFR_D_≤0.8 and 5 vessels were FFR_D_>0.8 among the vessels with invasive FFR≤0.8 in 32 vessels. Concerning FFR_CT_ calculated by the proposed approach, the numbers 4 and 50 represent that 4 vessels were calculated as having FFR_U_≤0.8, and 50 vessels were FFR_U_>0.8 among those with invasive FFR>0.8.

**TABLE 3:** The distribution of FFR_D_ and FFR_U_ calculated by the two approaches.

		Invasive FFR	
FFR_CT_ calculation		≤0.8	>0.8
FFR_D_ calculation	≤0.8	27	8
	>0.8	5	46
FFR_U_ calculation	≤0.8	30	4
	>0.8	2	50

### 3.4 Consistency evaluation of FFR_U_ and FFR_D_ with invasive FFR

**FIGURE 3:**
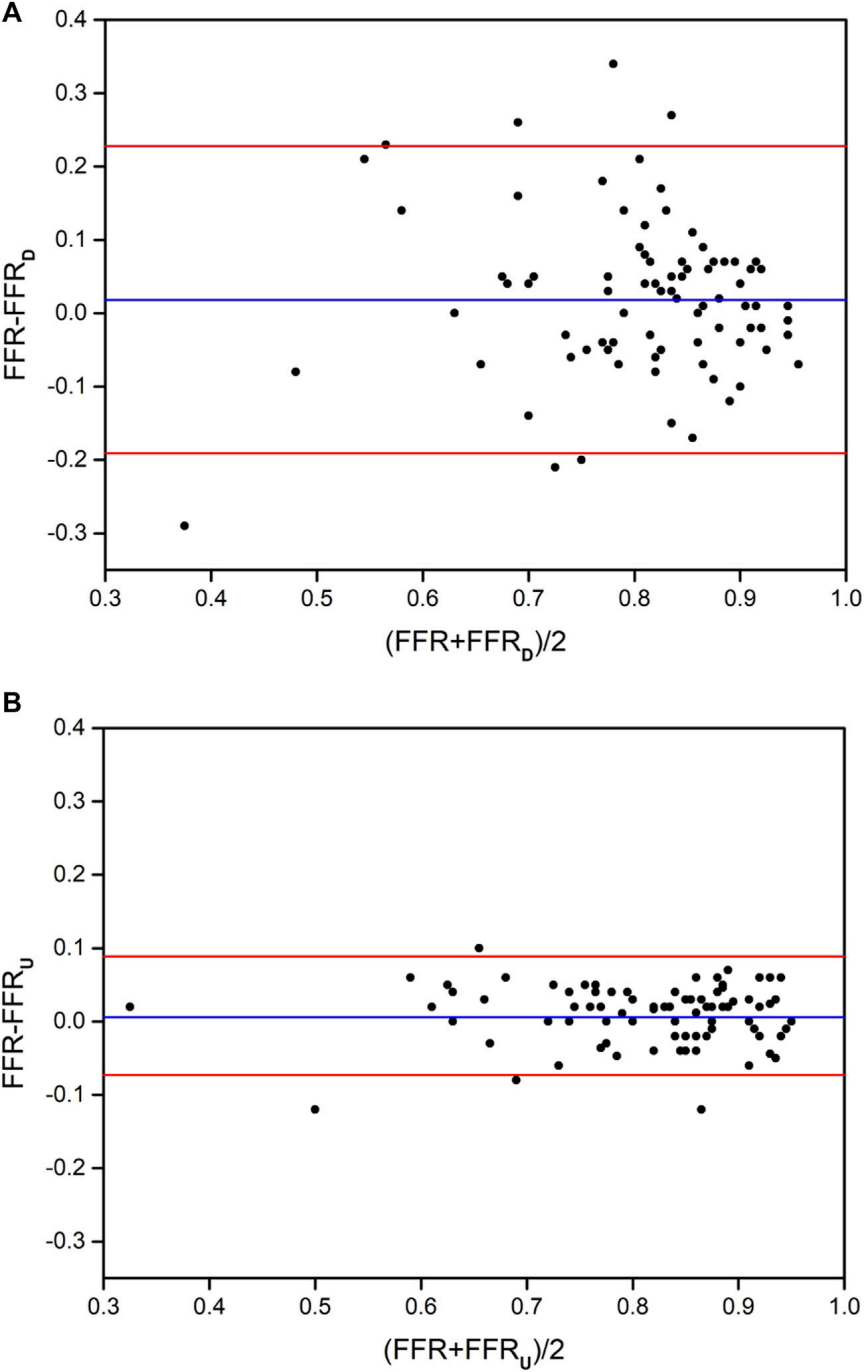
The distribution of Bland-Altman diagrams of FFR_D_ and FFR_U_. **(A)** the method of FFR_D_; **(B)** the method of FFR_U_.

Bland-Altman analysis was used to test the consistency of FFR_U_ and FFR_D_ with invasive FFR, respectively. The mean difference in FFR_U_-FFR (0.006) for all vessels was less than FFR_D_-FFR (0.018). The 95% CI of FFR_U_ and FFR_D_ was [-0.073, 0.085] and [-0.191, 0.228], respectively, and most of the data fell within the interval, indicating that FFR_U_ and FFR_D_ were in good agreement with invasive FFR, as shown in Figure 3.

### 3.5 Accuracy

**FIGURE 4:**
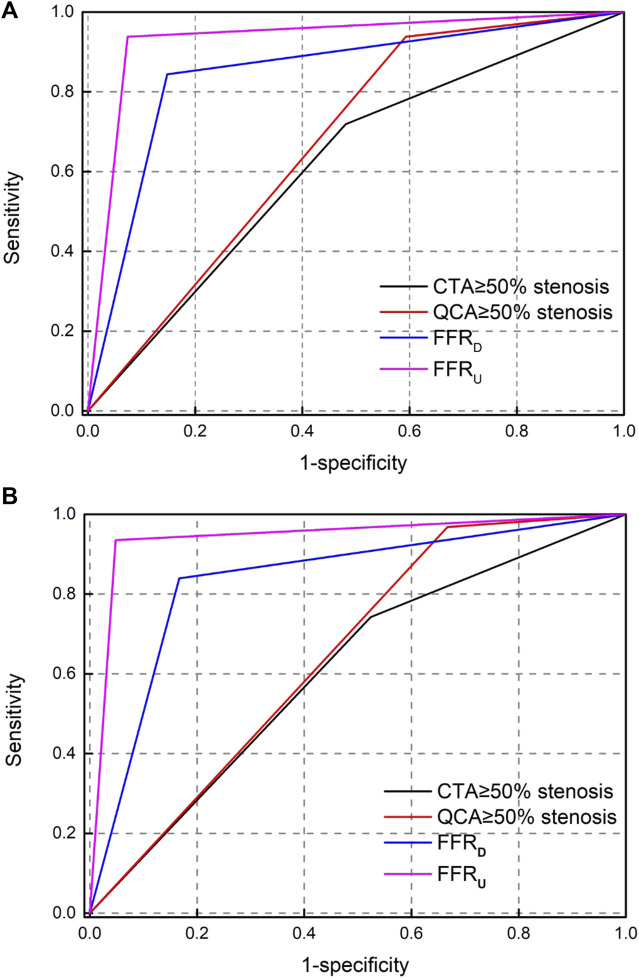
Receiver operating-characteristic (ROC) curve analysis for determining the area under the curve (AUC). **(A)** per-vessel level; **(B)** per-patient level.

The AUC of the receiver operating characteristics curve analysis at the lesion level for CTA, QCA, FFR_D_ and FFR_U_ were 0.62 (95% CI: 0.51–0.74), 0.67 (95% CI: 0.56–0.79), 0.85 (95% CI: 0.76–0.94), and 0.93 (95% CI: 0.87–0.98). At the patient level, the AUC was 0.61 (95% CI: 0.48–0.74) for CTA, 0.65 (95% CI: 0.53–0.77) for QCA, 0.83 (95% CI: 0.74–0.92) for FFR_D_, and 0.92 (95% CI: 0.89–0.96) for FFR_U_, as shown in Figure 4. The AUC at the lesion level demonstrated the diagnostic accuracy of FFR_U_ was higher than that of CTA, QCA, and FFR_D_. Similar results were observed at the patient level with FFR_U_ (AUC, 0.92) compared with CTA, QCA, and FFR_D_.

Table 4 shows that the per-vessel level sensitivity analysis of CTA, QCA, FFR_D_, and FFR_U_ were 71.87%, 93.75%, 84.38%, and 93.75%; specificity of 51.85%, 40.74%, 85.19%, and 92.59%; PPV of 46.94%, 48.39%, 77.14%, and 88.24%; and NPV of 75.68%, 91.67%, 90.2%, and 96.15%. The diagnostic accuracy of the four metrics of FFR_U_ was higher than that of CTA, QCA, and FFR_D_. Similar phenomena were observed at the patient level in FFR_U_ compared with CTA, QCA, and FFR_D_.

**TABLE 4:** The accuracy performance of CTA, QCA, FFR_D_, and FFR_U_ on the vessel and patient level.

Parameter	CTA≥50% stenosis	QCA≥50% stenosis	FFR_D_≤0.8	FFR_U_≤0.8
Per-vessel level				
Sensitivity (%)	71.87	93.75	84.38	93.75
Specificity (%)	51.85	40.74	85.19	92.59
PPV (%)	46.94	48.39	77.14	88.24
NPV (%)	75.68	91.67	90.2	96.15
Per-patient level				

	CTA≥50% stenosis	QCA≥50% stenosis	FFR_D_≤0.8	FFR_U_≤0.8
Sensitivity (%)	74.19	96.77	83.87	93.54
Specificity (%)	47.62	33.33	83.33	95.24
PPV (%)	51.11	51.72	78.79	93.55
NPV (%)	71.43	93.33	87.5	95.24

## 4 Discussion

In this study, we developed a novel method that integrated boundary condition settings with clinical statistics of hyperemia state, and the custom function took into account the interaction of stenotic resistance, microcirculation resistance, and inlet aortic pressure to identify coronary blood flow, further improving the accuracy of FFR_U_ calculation. Statistical results showed that the AUC of FFR_U_ calculation was 0.92 at the patient level and the computational time was 5 min per simulation, which was higher and faster than previous methods based on the same data. The main contributors to this study leading to higher diagnostic accuracy were: 1) the adoption of a 3D model instead of a 1D model to reflect the characteristics of stenotic structures, and 2) the adoption of the custom function to integrate the interaction of stenotic resistance, microcirculation resistance and inlet aortic pressure set based on boundary conditions of clinical statistics of hyperemia state.

A large number of clinical studies have shown that FFR_CT_ calculation had certain limitations when only a 1D model of coronary artery considering diameter stenosis and stenotic length was used for CFD simulation (Fossan et al., 2018; Ge et al., 2019; Müller et al., 2019). The accuracy of FFR_CT_ calculation was insufficient to be applied when compared with the method proposed by Taylor (84.3%). In this study, our method adopted a 3D model instead of a 1D model to reflect the characteristics of stenotic structures in CFD simulation to improve the accuracy of FFR_CT_ calculation. Based on the 3D model, the detailed characteristics of coronary stenotic structures were systematically and comprehensively considered and applied to FFR_CT_ calculation. The advantage of using a 3D model was that the pressure distribution of coronary arteries can be calculated intuitively and accurately, which depended on the frictional head loss caused by the coronary distribution, as well as the local head loss caused by the structural characteristics of the stenosis (especially irregular geometric). The 3D model can deeply analyze hemodynamic parameters such as pressure and blood flow at any axial and radial positions of the model, and evaluate the impact of local geometric structure on FFR_CT_ calculation from a qualitative or quantitative perspective. The FFR_CT_ calculation mainly explored the impact of the local geometric structure of the coronary stenotic structure on the pressure distribution. Therefore, the 3D model should be considered to improve the diagnostic accuracy of FFR_CT_ calculation.

Many studies have proved that the boundary condition settings had a significant impact on the accuracy of FFR_CT_ calculation (Sankaran et al., 2016; Ernest et al., 2020; Liu et al., 2020). Previous studies have explored the boundary condition settings based on clinical statistics of hyperemia state to improve the accuracy of FFR_CT_ calculation (Nørgaard et al., 2014; Zhang et al., 2021b; Chandola et al., 2021). However, the boundary condition settings of these studies ignored the interaction of stenotic resistance, microcirculation resistance, and inlet aortic pressure to improve the accuracy of FFR_CT_ calculation. In this study, based on clinical statistics of hyperemia state, we developed a novel method to couple the interaction of stenotic resistance, microcirculation resistance, and inlet aortic pressure by loading the custom function for the boundary condition settings of FFR_U_ calculation. Based on the custom function, the boundary condition settings and FFR_U_ calculation were carried out in an individual, systematic, and integrated manner. The advantage of using the custom function was to quantitatively analyze the effect of stenotic resistance, microcirculation resistance, and inlet aortic pressure on the blood flow set by the outlet boundary condition, so as to accurately and rapidly identify coronary blood flow, and this further improved the accuracy of FFR_U_ calculation. Consequently, based on the boundary condition settings using the custom function, our proposed method can accurately and rapidly identify coronary blood flow, enabling digital non-invasive assessment of myocardial ischemia caused by coronary stenosis. The results of improved levels of the accuracy of FFR_U_ calculation, compared with previous methods, indicate that our proposed method can be used as a reference index for the diagnosis of myocardial ischemia caused by coronary stenosis in clinical practice.

### 4.1 Limitations and future work

Although the valuable information derived from our novel method, improved the diagnostic accuracy and reduced the computational time for FFR_U_ calculation, several limitations are notable. First, 73 patients undergoing CTA, QCA, and invasive FFR were enrolled from two central databases. The diversity and number of patients were relatively small. So the diversity and number of patients are enrolled from multiple centers in our future work. Second, the compliance of the epicardial coronary artery was neglected from resting to hyperemia state, and some studies had reported that the compliance of the epicardial coronary artery had almost no difference in the changes of coronary blood flow and pressure (Zeng et al., 2008; Zhang et al., 2014). Third, mean blood pressure was reduced by 12% to mimic the mean blood pressure changes from resting to hyperemia state in all patients. It ignored the effect of patient-special mean blood pressure changes on FFR_U_ calculation, and Zhang et al. reported that the mean blood pressure changes had almost no difference for FFR_CT_ calculation (Zhang et al., 2020). Finally, the steady-state numerical simulation employed in this study calculated the FFR_U_, ignoring the effect of the pulsatile flow characteristics on FFR_U_ calculation. And the pulsatile flow was reported to be less important in FFR_CT_ calculation (Zhang et al., 2014).

## 5 Conclusion

In this study, we developed a novel method for FFR_U_ calculation using the custom function. Based on the comparison results of existing methods and FFR_U_ calculation with invasive FFR, our proposed method can accurately and rapidly identify coronary blood flow, significantly improving the accuracy of FFR_CT_ calculation. This study indicates that the proposed novel method might realize digital non-invasive evaluation of myocardial ischemia caused by coronary stenosis, supporting its wide clinical application in the diagnosis of myocardial ischemia caused by coronary stenosis.

## Data Availability

The original contributions presented in the study are included in the article/Supplementary material, further inquiries can be directed to the corresponding author.
